# Medical Thermometry and Human Temperature

**Published:** 1876-05

**Authors:** 


A Manual of General Pathology. By Ernst Wagner, M.D., Professor of General Pathology and Pathological Anatomy University of Leipzig. Translated from the sixth German edition, by John Van Duyn, A.M., M.D., Professor of Anatomy and Histology in Medical School, Syracuse, N. Y., and E. C. Seguin, M.D., Clinical Professor of Diseases of Mind and Nervous System, College of Physicians and Surgeons, N. J. New York: William Wood & Co. 1876. Book, pp. 727.

This work is composed of four divisions. Part first treats of general nosology; second, general ætiology; third, general pathological anatomy and physiology; fourth, pathology of blood.

Upon a hasty perusal of this work, we are struck with the able manner in which the different parts are investigated and made practical. It is the duty of the profession to search after truth to the very utmost, and as a guide to pathological anatomy we recommend the above work.

---

Medical Thermometry and Human Temperature. By E. Seguin, M.D. Book, pp. 446. New York: Wm. Wood & Co., 37 Great Jones street. 1876.

A large portion of this work is devoted to the temperature of the body in both health and disease, giving in detail the exacerbations of heat in the various diseases; also, the difference between infantile and adult age, and male and female. The author discusses at some length the thermometer and its various uses, etc. It is a work of 446 pages, neatly arranged and bound, and for sale by Wm. Wood & Co., New York.

